# KDM4C silencing inhibits cell migration and enhances radiosensitivity by inducing CXCL2 transcription in hepatocellular carcinoma

**DOI:** 10.1038/s41420-023-01418-w

**Published:** 2023-04-28

**Authors:** Zhen Zeng, Zixuan Li, Jun Xue, Huichan Xue, Zhiwei Liu, Wenxuan Zhang, Hongli Liu, Shuangbing Xu

**Affiliations:** 1 Cancer Center, Union Hospital, Tongji Medical College, Huazhong University of Science and Technology, 430022 Wuhan, China; 2 Institute of Radiation Oncology, Union Hospital, Tongji Medical College, Huazhong University of Science and Technology, 430022 Wuhan, China

**Keywords:** Hepatocellular carcinoma, Radiotherapy

## Abstract

KDM4C, which is a histone lysine demethylase, has been proposed to participate in the malignant transformation and progression of several types of cancer. However, its roles in hepatocellular carcinoma (HCC) remain poorly understood. Here, we find that KDM4C protein expression is increased in HCC and promotes HCC cell growth, proliferation and migration. Furthermore, we provide evidence that depletion of KDM4C leads to a defective G2/M checkpoint, increases radiation-induced DNA damage, impairs DNA repair and enhances radiosensitivity in HCC cells. Using RNA sequencing, we identify that the chemokine CXCL2 is a downstream effector of KDM4C. KDM4C knockdown increases the binding of H3K36me3 to the promoter of CXCL2, thus upregulating CXCL2 expression and promoting CXCL2 secretion in HCC cells. Importantly, the observed effects of KDM4C depletion in HCC cells can be partially rescued by CXCL2 silencing. Thus, our findings reveal that KDM4C is involved in cell migration and radiosensitivity by modulating CXCL2 transcription, indicating that KDM4C may be a potential therapeutic target in HCC.

## Introduction

Primary liver cancer, which is one of the most prevalent cancers, ranks third among the causes of cancer-related death, and it has been a challenge that threatens human health worldwide [1, 2]. Hepatocellular carcinoma (HCC), which is the most common type of primary liver cancer, accounts for 75–85% of primary liver cancer cases [1]. At present, hepatic resection, transarterial therapies, radiotherapy and systemic therapies are the main treatments for HCC [3]. However, because HCC is often initially diagnosed at an advanced stage, the mortality rate of HCC is still high [4]. Therefore, the discovery of new diagnostic and prognostic biomarkers is urgently needed to improve the survival rate of HCC patients.

Cancer progression is a complicated process that involves multiple genetic mutations and epigenetic modifications [5, 6]. Of these factors, epigenetics is increasingly being shown to be important for cancer development and progression [7]. Epigenetic regulation mainly includes DNA methylation and histone post-translational modifications; of these, histone methylation plays a vital role in cancer [7]. Histone lysine demethylase 4 °C (KDM4C), which belongs to the jumonji domain containing 2 (JMJD2) subfamily, encodes a protein that functions as a di-/tri-methylation demethylase that targets lysine 9/36 on histone 3 to control downstream gene expression [8]. For instance, KDM4C has been reported to regulate the expression of NOTCHI1 and the self-renewal of stem cells in breast cancer [9]. It has been demonstrated that KDM4C inhibits p53 activation and conversely activates c-Myc to induce apoptosis in glioblastoma [10]. In addition, growing evidence has reported that KDM4C is overexpressed and promotes tumorigenesis in multiple types of human cancer [11–15]. Our recent studies revealed crucial roles of KDM4C in lung cancer radioresistance and antitumor immunity [16, 17]. Nevertheless, whether KDM4C is functionally correlated with HCC has not yet been fully elucidated.

Here, we reveal that KDM4C is upregulated in HCC cells and promotes cell growth and proliferation. More importantly, we present evidence that targeting KDM4C inhibits the migration and enhances the radiosensitivity of HCC cells. Mechanistically, we show that KDM4C exerts its biological effects by regulating CXCL2 expression. These results illustrate that KDM4C might be a promising therapeutic target for the treatment of HCC.

## Results

### KDM4C protein is highly expressed and promotes the growth, proliferation and migration of HCC cells

**Fig. 1:**
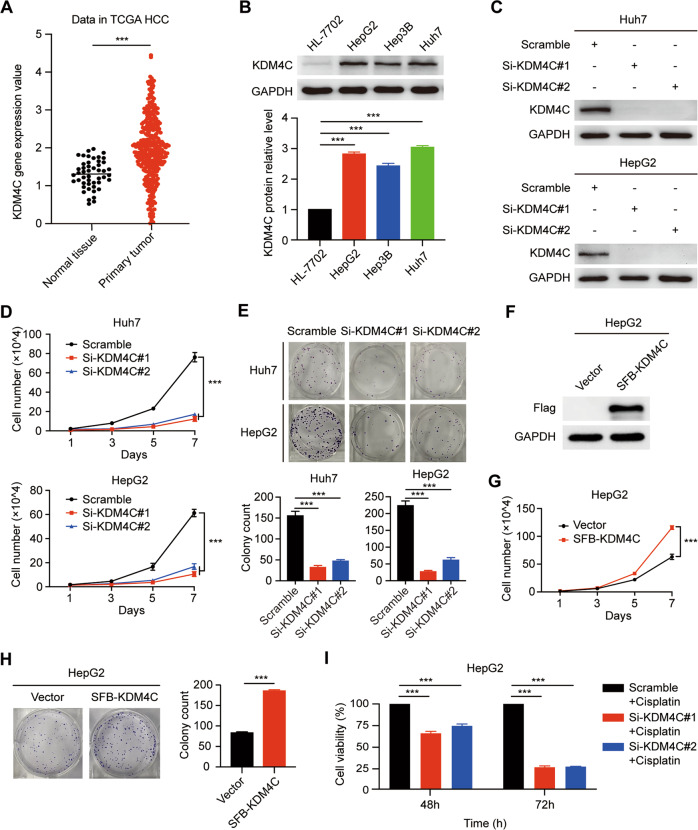
KDM4C is upregulated in HCC and promotes cell growth and proliferation. **A** KDM4C was upregulated in primary HCC tumor tissue than in normal tissue in TCGA dataset. **B** Upper panel: the protein expression levels of KDM4C in several cell lines were measured by western blotting. Lower panel: the gray values of the protein bands were quantified using ImageJ software (*n* = 3). **C** KDM4C was effectively knocked down in Huh7 and HepG2 cells using the two indicated siRNAs (*n* = 3). **D** Cells were transfected with the indicated siRNAs, seeded in six-well plates and counted every other day (*n* = 3). **E** Cells transfected with the indicated siRNAs were plated and cultured in six-well plates (500 cells/well) for 2 weeks followed by fixation and staining. Colony (>50 cells) numbers were counted (*n* = 3). **F** HepG2 cells transfected with SFB-tagged KDM4C were lysed and analyzed by western blotting (*n* = 3). **G**, **H** Overexpression of KDM4C promoted cell growth and proliferation in HepG2 cells (*n* = 3). **I** HepG2 cells were transfected with the indicated siRNAs for 24 h and then treated with 0.6 μM cisplatin. The percentage of cell viability was measured after 48 and 72 h (*n* = 3).

**Fig. 2:**
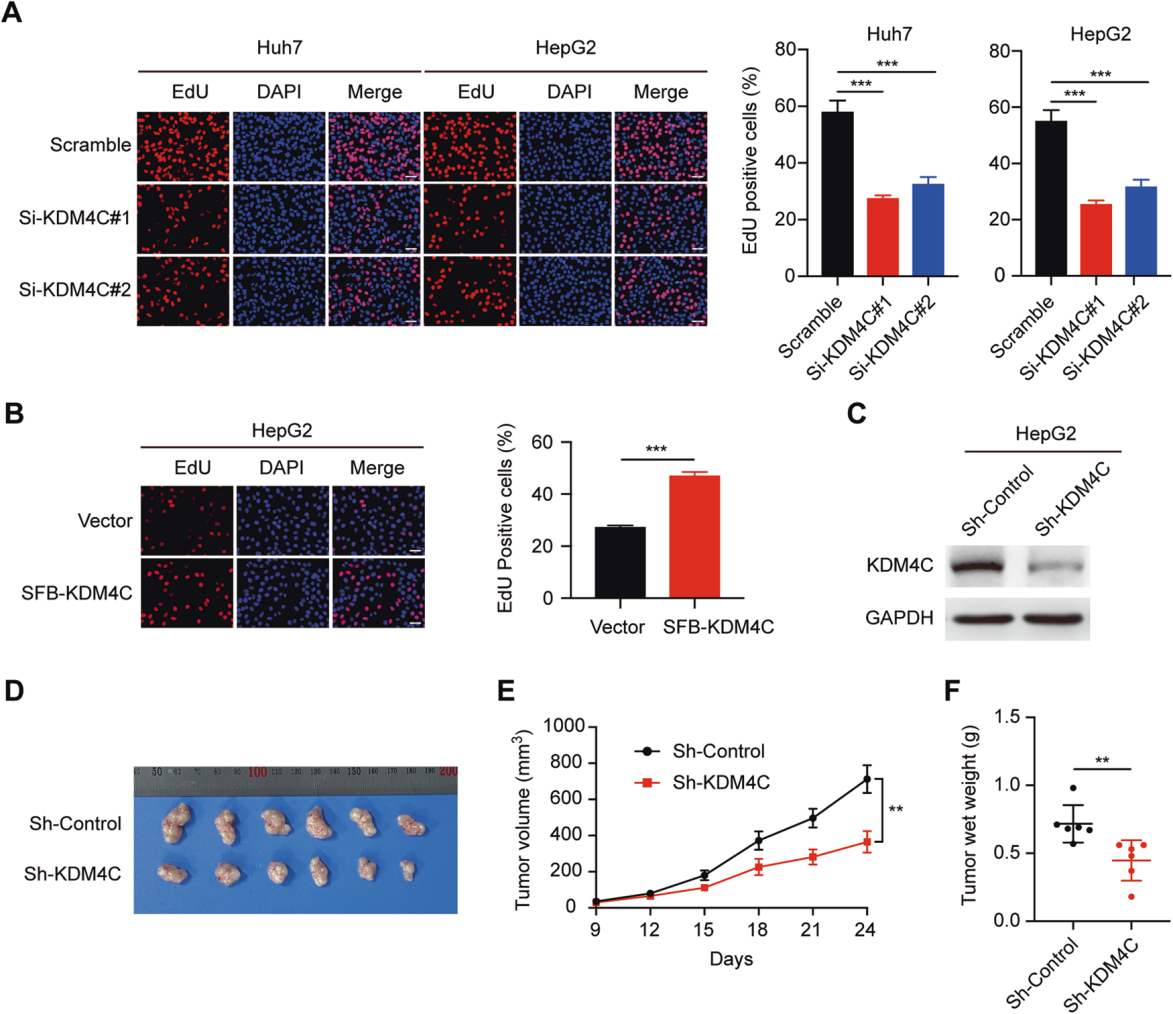
KDM4C knockdown inhibits HCC tumor growth in vivo. **A**, **B** Left panel: cell proliferation ability was examined by EdU assay. Right panel: the Edu-positive cell rate in each group was calculated (*n* = 3). Scale bar, 50 μm. **C** KDM4C was effectively knocked down using KDM4C-targeting shRNAs in HepG2 cells (*n* = 3). **D** Images of subcutaneous xenograft tumors (six mice/group). **E** The tumor volume was measured and calculated every three days (six mice/group). **F** The wet weight of the tumors (six mice/group).

To explore the role of KDM4C in HCC, we first analyzed KDM4C mRNA expression in HCC and normal tissues using TCGA database. As shown in Fig. 1A, KDM4C mRNA levels were upregulated in HCC tissues compared with normal tissues. Next, we measured the protein level of KDM4C in human HL-7702 normal liver cells and several hepatocellular carcinoma cell lines. As shown in Fig. 1B, KDM4C was highly expressed in the HepG2, Hep3B and Huh7 cell lines compared to the HL-7702 cell line, indicating that KDM4C may function as an oncoprotein in HCC. Consequently, we used two siRNAs that target KDM4C to silence its expression in HepG2 and Huh7 cells for further functional analysis (Fig. 1C). As shown in Fig. 1D and E, cell growth and proliferation were significantly attenuated by KDM4C silencing. Consistent with this idea, overexpression of KDM4C dramatically promoted HCC cell growth and proliferation (Fig. 1F–H). Furthermore, we found that KDM4C silencing also enhanced the chemosensitivity of cisplatin in HCC cells (Fig. 1I). In addition, the EdU assay showed that the percentage of EdU-positive cells was notably decreased in KDM4C-depleted cells compared with that of control cells whereas overexpression of KDM4C exhibited the opposite effect (Fig. 2A and B). Importantly, using the xenograft model, we demonstrated that both tumor size and weight of mice injected with KDM4C stable knockdown HCC cells were decreased compared with those of mice injected with control HepG2 cells (Fig. 2C–F). These results support the notion that KDM4C facilitates tumorigenesis in HCC in vitro and in vivo.

**Fig. 3:**
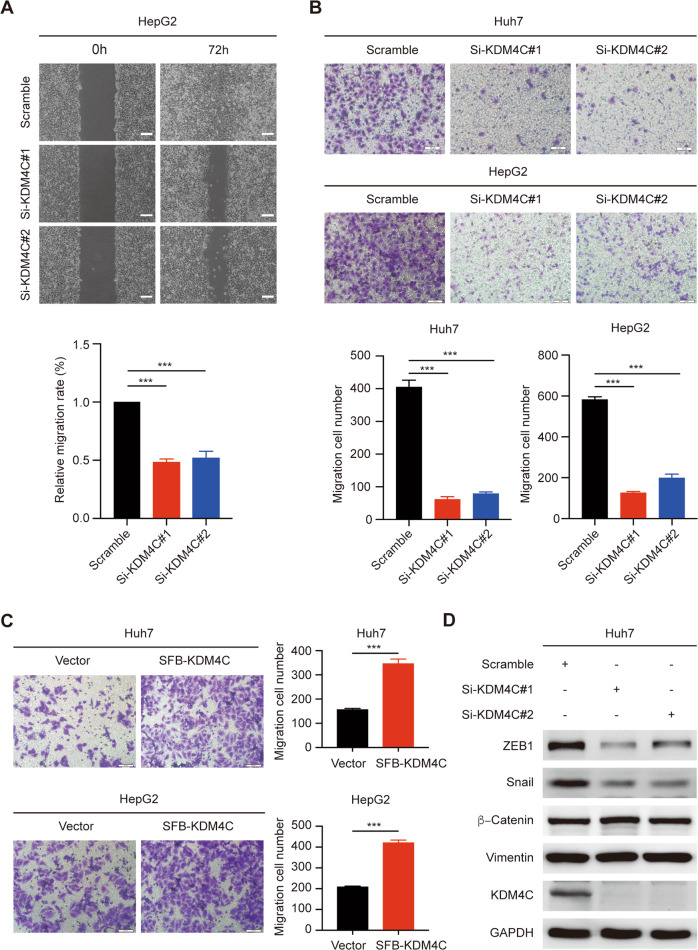
Inhibition of KDM4C suppresses HCC cell migration. **A** Upper panel: the confluent monolayer of HepG2 cells in each group was scratched and cultured for an additional 72 h to measure the wound-healing rate. Lower panel: the relative area migration rate was calculated (*n* = 3). **B**, **C** Transwell assays were performed to evaluate the migration of Huh7 and HepG2 cells. Quantification of the migrated cell number is shown in histograms (*n* = 3). **D** Huh7 cells were transfected with KDM4C siRNAs for 48 h and then collected and analyzed by western blotting (*n* = 3).

To further investigate the effect of KDM4C on the migration of HCC cells, a wound-healing assay was performed. As shown in Fig. 3A, the rate of wound healing was slower in KDM4C-knockdown cells than in control cells. In accordance with this result, the Transwell assay revealed decreased cell migration when KDM4C was depleted (Fig. 3B). In contrast, overexpression of KDM4C enhanced the migration ability of HCC cells (Fig. 3C). Consistent with these results, KDM4C silencing downregulated the protein levels of migration markers ZEB1 and Snail, whereas the levels of β-catenin and vimentin were not altered (Fig. 3D). Together, these findings suggest that targeting KDM4C inhibits HCC cell migration.

### KDM4C depletion enhances radiosensitivity in HCC cells

**Fig. 4:**
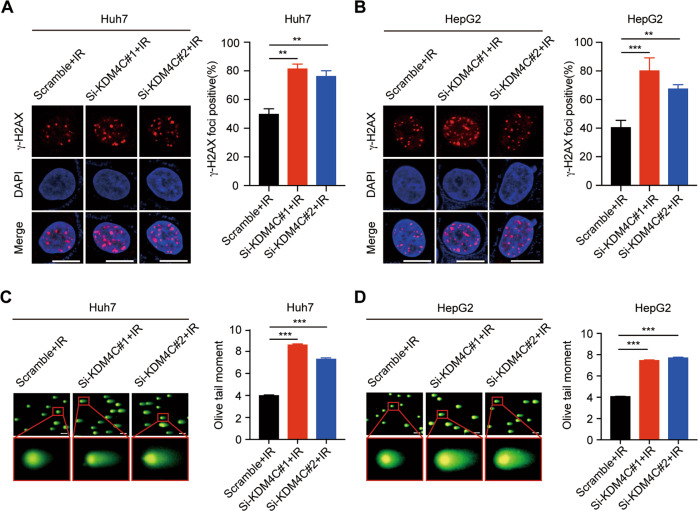
Knockdown of KDM4C enhances IR-induced DNA damage in HCC cells. **A**, **B** Immunofluorescence staining of γ-H2AX foci was conducted to measure the effect of KDM4C depletion on IR-induced DNA damage in Huh7 and HepG2 cells. Representative images and γ-H2AX-positive cell quantification are shown (*n* = 3). Scale bar, 10 μm. **C**, **D** A neutral comet assay was carried out in Huh7 and HepG2 cells to assess single-cell DNA damage post-irradiation in each group. Images were captured under an immunofluorescence microscope, and the Olive Tail Moment was calculated using CometScore2.0 is presented (*n* = 3). Scale bar, 50 μm.

To test whether KDM4C is involved in HCC radiosensitivity, we first used immunofluorescence staining to assess γ-H2AX foci formation. As expected, loss of KDM4C expression increased the γ-H2AX-positive rate after 6 Gy IR exposure (Fig. 4A and B), indicating that knockdown of KDM4C augmented IR-induced DNA damage. Furthermore, we analyzed the single-cell DNA double-strand breaks (DSBs) caused by 6 Gy irradiation via a neutral comet assay. As shown in Fig. 4C and D, an increase in the Olive tail moment was observed in KDM4C-depleted cells. These data confirm that KDM4C silencing promotes DNA damage in HCC cells.

**Fig. 5:**
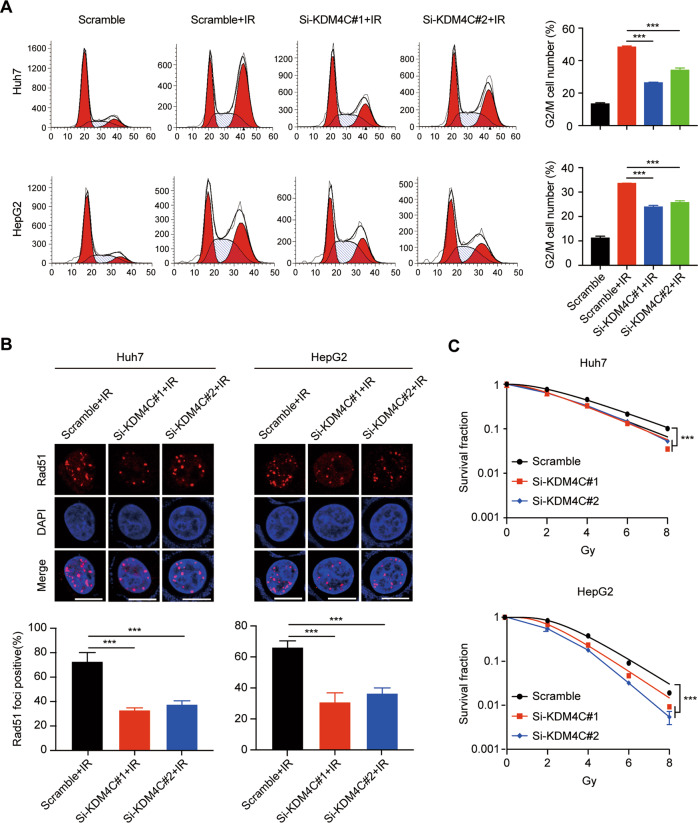
Loss of KDM4C inhibits IR-induced DNA damage repair and enhances radiosensitivity in HCC cells. **A** Huh7 and HepG2 cells were treated as indicated, and the proportions of cells in each phase of the cell cycle were analyzed using ModFit software. Results are shown in histograms (*n* = 3). **B** KDM4C silencing led to reduced rad51 foci formation by Huh7 and HepG2 cells 4 h post-irradiation. Representative immunofluorescence images and statistical diagrams are shown (*n* = 3). Scale bar, 10 μm. **C** Knockdown of KDM4C contributed to enhanced radiosensitivity in HCC cells (*n* = 3).

Considering that cells that are damaged by irradiation undergo cell cycle arrest, which is an important mechanism of cell protection that prevents cell death by repairing damaged DNA, we conducted a cell cycle assay and found that after IR treatment at 6 Gy, the number of cells in the G2/M phases was significantly increased, and this effect was partially abolished by KDM4C depletion (Fig. 5A). As Rad51 plays a central role in homologous recombination repair, we examined Rad51 foci formation and found that the proportion of Rad51-positive cells was decreased when KDM4C was depleted (Fig. 5B), suggesting that depletion of KDM4C expression impairs DNA repair. Consistent with this notion, the clonogenic survival assay showed that KDM4C knockdown enhanced cell sensitivity to irradiation (Fig. 5C). Taken together, these results strongly suggest that KDM4C silencing enhances radiosensitivity in HCC cells.

### KDM4C silencing induces CXCL2 transcription and promotes its secretion in HCC cells

**Fig. 6:**
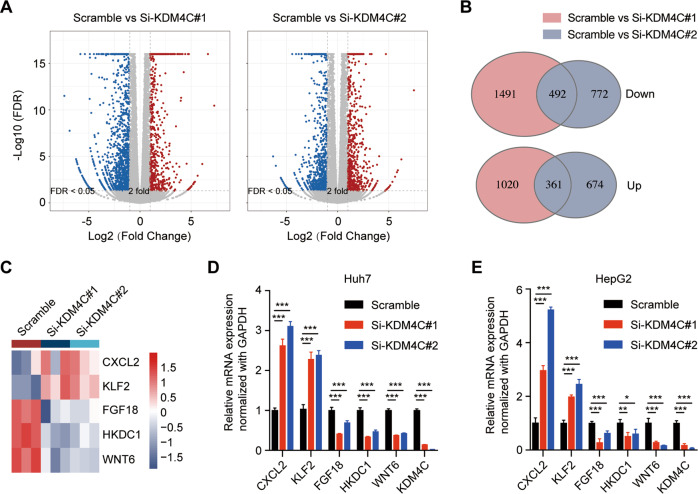
KDM4C silencing upregulates CXCL2 mRNA expression in HCC cells. **A** Volcano maps depicting the differentially expressed genes in KDM4C-depleted Huh7 cells compared with Scramble-transfected cells. **B** Venn diagrams showing co-downregulated and co-upregulated genes. **C** Heatmap showing screened genes related to HCC. **D**, **E** Relative mRNA expression levels of genes in Huh7 and HepG2 cells transfected with the indicated siRNAs were measured using quantitative real-time PCR (*n* = 3).

To elucidate the downstream mechanisms by which KDM4C functions in these processes, we carried out RNA sequencing analysis in Huh7 cells. Differentially expressed genes that met the criteria of false discovery rate (FDR) < 0.05 and fold change (Fc) >2 were plotted as blue and red dots in volcano maps, representing downregulated and upregulated genes, respectively (Fig. 6A). We found that there were 492 co-downregulated genes and 361 co-upregulated genes, as shown in the Venn diagrams (Fig. 6B).

**Fig. 7:**
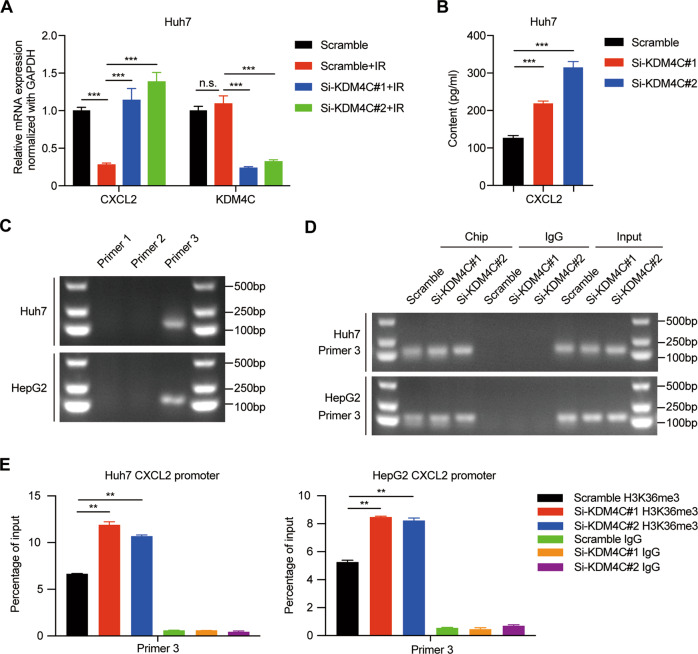
KDM4C silencing increases H3K36me3 accumulation at the CXCL2 promoters and induces CXCL2 expression in HCC cells. **A** Relative mRNA expression levels of CXCL2 and KDM4C in HepG2 cells with or without irradiation (*n* = 3). **B** CXCL2 secretion protein levels were measured using ELISA (*n* = 3). **C**, **D** Representative images of gel electrophoresis from the ChIP assay (*n* = 3). **E** The percentage of H3K36me3 at the CXCL2 promoter region was normalized to the input and quantified (*n* = 3).

Given that our study focused on HCC, we concentrated mainly on genes that are involved in HCC. CXCL2, KLF2, FGF18, HKDC1, and WNT6 have been reported to be involved in HCC [18–22], and these genes are listed in the heatmap (Fig. 6C). We further confirmed the mRNA expression levels of these genes in KDM4C-depleted cells using a real-time PCR assay. As shown in Fig. 6D and E, CXCL2 and KLF2 were upregulated, whereas FGF18, HKDC1, and WNT6 were downregulated when KDM4C was knocked down in both Huh7 and HepG2 cells. Notably, CXCL2 was the most significantly changed gene (Fig. 6D and E), and its mRNA levels were increased both under normal conditions and in response to DNA damage when KDM4C was depleted (Fig. 7A). Additionally, the ELISA also demonstrated that KDM4C silencing induced the secretion of CXCL2 by HCC cells (Fig. 7B). It has been well documented that the decrease of H3K36me3 mediated by KDM4C results in transcriptional inactivation of downstream genes [23]. To further elucidate how CXCL2 is regulated by KDM4C, we performed the ChIP PCR assay and showed that KDM4C silencing increased the binding affinity of H3K36me3 for the CXCL2 gene promoter region (Fig. 7C–E). These data suggest that KDM4C can induce the expression of CXCL2 by promoting the accumulation of H3K36me3 at the CXCL2 promoter in HCC cells.

### The biological functions of KDM4C in HCC are partially dependent on CXCL2

**Fig. 8:**
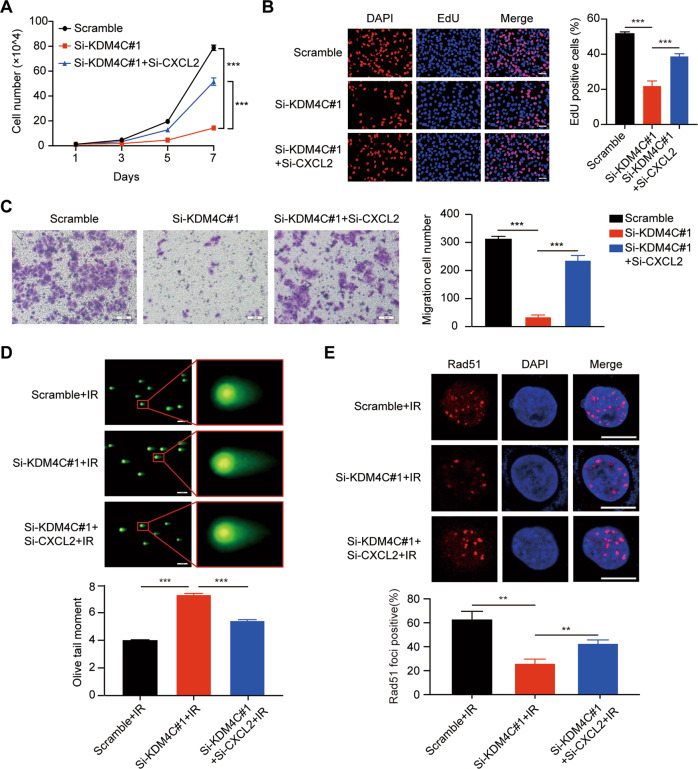
KDM4C exerts its biological effects by regulating CXCL2 expression in Huh7 cells. **A** Huh7 cells were transfected with the indicated siRNAs, seeded in six-well plates and counted every other day (*n* = 3). **B** Cell proliferation was examined by an EdU assay (*n* = 3). Scale bar, 50 μm. **C** Transwell assays were performed to evaluate the migration of Huh7 cells (*n* = 3). Scale bar, 50 μm. **D** A neutral comet assay was conducted in Huh7 cells to assess single-cell post-irradiation DNA damage in each group (*n* = 3). Scale bar, 50 μm. **E** Rad51 foci formation was used to analyze DNA repair. Representative immunofluorescence images and statistical diagrams are shown (*n* = 3). Scale bar, 10 μm.

To determine whether CXCL2 is a downstream effector of KDM4C, rescue experiments were conducted in HCC cells. As shown in Fig. 8A and B, the reduced growth and proliferation caused by KDM4C depletion could be partially restored by simultaneously inhibiting CXCL2. Moreover, Transwell assays showed that the suppression of cell migration by KDM4C inhibition was reversed by CXCL2 depletion (Fig. 8C). Similarly, the enhanced DNA damage and impaired DNA repair that was caused by KDM4C knockdown were also partially rescued by transfection with siRNAs that target CXCL2 (Fig. 8D and E). All of these results confirm that the biological functions of KDM4C in HCC are partially dependent on CXCL2.

## Discussion

In this study, we present evidence that the depletion of KDM4C inhibits cell proliferation and migration and enhances radiosensitivity in HCC cells. Moreover, we showed the chemokine CXCL2 as a critical downstream effector of KDM4C that mediates the biological effects of KDM4C, indicating that targeting the KDM4C/CXCL2 axis may be a promising therapeutic strategy for HCC treatment.

It has been documented that KDM4C is overexpressed and that KDM4C dysregulation is correlated with tumorigenesis, B-cell activation and amino acid metabolism [24–26]. For example, KDM4C can induce the transcription of Wnt/β-catenin target genes and promote glioblastoma tumorigenesis [27]. Knockout of KDM4C suppresses glycolytic metabolism to inhibit prostate cancer metastasis by inhibiting C-Myc/LDHA signaling [28]. However, few studies have explored the functions of KDM4C in HCC. Our results demonstrate that KDM4C is upregulated in HCC cells and drives cell growth and proliferation in vitro and in vivo, which is consistent with a recent study showing that KDM4C knockdown led to decreased HCC proliferation by stabilizing ROCK2 [29]. In addition, we confirmed for the first time that KDM4C is inversely correlated with the migration of HCC cells. More importantly, our study also provides a novel finding that KDM4C depletion causes IR-induced DNA double-strand breaks and abolishes G2/M checkpoint arrest, therefore resulting in reduced DNA repair and enhanced radiosensitivity in HCC cells.

C-X-C motif chemokine ligand 2 (CXCL2), which belongs to the CXC chemokine family, is a small secreted protein that participates in cancer progression and metastasis, osteoblast differentiation and immune regulation [30–33]. Ding et al. demonstrated that CXCL2 functions as a tumor suppressor to inhibit proliferation and promote apoptosis by modulating the ERK1 signaling pathway in HCC cells [18]. Recent work has shown that CXCL2 can recruit neutrophils to induce immunosuppressive phenotypes in HCC [34]. In our study, we performed RNA sequencing and identified CXCL2 as a critical effector of KDM4C in HCC cells. Inhibition of KDM4C upregulates the expression of CXCL2 at both the transcriptional and translational levels, and this regulation is dependent on the demethylase activity of KDM4C by promoting the binding of H3K36me3 to the CXCL2 promoter. Notably, the mRNA level of CXCL2 is downregulated after irradiation, indicating that CXCL2 may be a DNA damage-responsive gene, although this warrants further investigation in future. In addition, our results clearly show that the phenotypes caused by KDM4C silencing are partially dependent on CXCL2 expression. Therefore, this work reveals that CXCL2 is a novel downstream effector of KDM4C and provides new insights into the mechanism by which KDM4C functions in HCC cells.

In summary, our work confirms the critical roles of KDM4C in the proliferation, migration, and radiosensitivity of HCC cells by upregulating the expression of CXCL2. These findings indicate that KDM4C might be a prospective biomarker for HCC diagnosis and treatment.

## Materials and methods

### Cell culture and plasmid transfection

The human HL-7702 liver cell line, hepatocellular carcinoma cell lines (HepG2, Huh7, and Hep3B) and human embryonic kidney cell line HEK-293T were obtained from American Type Culture Collection (ATCC) and maintained in DMEM supplemented with 10% FBS and 1% penicillin‒streptomycin solution in an incubator at 37 °C in 5% CO_2_. These cell lines were authenticated by STR profiling and tested for mycoplasma contamination. The vector and SFB-KDM4C plasmids were transfected into Huh7 and HepG2 cells using Lipofectamine 2000 reagent (Invitrogen, USA).

### RNA interference

Small interfering RNA (siRNA) transfection was performed using Lipofectamine RNAiMAX reagent (Invitrogen, USA). Forty-eight hours post-transfection, the cells were collected and used for further experiments. For the knockdown of KDM4C and CXCL2, the following siRNA oligonucleotides were used:

Si-KDM4C#1: 5’-AGAUAGCAGCAAUGAAGAA-3’ [16]

Si-KDM4C#2: 5’-GCTGGAGAATTCATGATCA-3’

Si-CXCL2: 5’-GGGCAGAAAGCTTGTCTCA-3’ [18]

### Establishment of KDM4C stable knockdown cells

Short hairpin RNA (shRNA) and pSPAX2 and pMD2G were transfected into HEK-293T cells to generate supernatants that were used to infect HepG2 cells with the addition of 10 mg/mL polybrene (Sigma–Aldrich, USA). KDM4C stable knockdown cells were selected using 2 mg/mL puromycin, and the KDM4C protein level was confirmed by western blotting. The sequences of the shRNAs used are listed below:

Sh-Control: 5’-CCTAAGGTTAAGTCGCCCTCG-3’

Sh-KDM4C: 5’-CCTTGCATACATGGAGTCTAA-3’

### Western blotting analysis

The cells were lysed in 1× RIPA lysis buffer to acquire the total cellular proteins. The supernatants were collected and then subjected to western blotting analysis. In brief, the proteins were first separated by SDS-PAGE and then transferred to 0.2 mm PVDF membranes, which were blocked for 1 h. The membranes were then incubated with specific primary antibodies overnight and subsequently with secondary antibodies for 1 h. The protein bands were detected using the Enhanced chemiluminescent (ECL) detection reagent (Bio-Rad, USA). The antibodies used in this study were as follows: rabbit anti-KDM4C (Bethyl Laboratories, A300-885A, 1:1000, USA); rabbit anti-GAPDH (Servicebio, GB11002, 1:1000, China); mouse anti-Flag (ABclonal, AE005, 1:1000, China); and rabbit anti-ZEB1, anti-Snail, anti-β-Catenin, anti-Vimentin (Cell Signaling Technology, #9782, USA).

### RNA sequencing and analysis

Huh7 cells were transfected with two siRNAs that target KDM4C or the scramble control. Total cellular RNA was isolated using TRIzol reagent (Invitrogen) and sent to CapitalBio Technology (Beijing, China) for RNA sequencing. After quantifying the final libraries, the data were subsequently sent to an Illumina NovaSeq sequencer (Illumina) for paired-end sequencing. Data that met the requirements of false discovery rate (FDR) < 0.05 and fold change (Fc) > 2 were used for analysis. All the data were deposited into the Gene Expression Omnibus database (GEO, GSE189941).

### Quantitative real-time PCR

Cellular RNA was extracted using a Total RNA kit I (Omega Bio-Tek, R6834-01, USA) in accordance with the manufacturer’s instructions. Reverse transcription was performed to synthesize cDNA, which was later used for quantitative real-time PCR. The ΔΔCt method was used to analyze the normalized mRNA levels of the target genes. The primer sequences used in this study are listed in Supplementary Table 1.

### Chromatin immunoprecipitation (ChIP) assay

This assay was carried out using a ChIP Assay Kit (Beyotime, P2078) according to the manufacturer’s instructions. Briefly, cells were collected after being fixed with 1% methanal and then broken up using an ultrasonic processor. The samples were mixed with IgG/H3K36me3 antibody (ABclonal, A20379) and protein A/G agarose overnight at 4 °C. RT-PCR was performed to detect H3K36me3 accumulation at the CXCL2 promoter region. The primer sequences are listed in Supplementary Table 2.

### Enzyme-linked immunosorbent assays (ELISAs)

The Human CXCL2/GRO-beta ELISA Kit (ABclonal, RK00150, China) was used for the quantification of CXCL2 levels according to the manufacturer’s instructions. Briefly, sample/standard was added to 96-well plates that were pre-coated with a capture antibody targeting CXCL2. After 2 h of incubation, the biotin-conjugated CXCL2 antibody was added and incubated for 1 h to bind the antigen-antibody complexes, followed by streptavidin-HRP attachment for 30 min. The complexes were then incubated with TMB substrates in the dark for 20 min, and the stop solution was added to terminate the reaction. OD values were measured at 450 nm with an enzyme-labeled colorimeter.

### Cell growth and colony formation assays

For the cell growth assay, cells were plated in six-well plates (1 × 10^4^ cells/well) in triplicate. Cell numbers were counted at 1, 3, 5, and 7 days. For the cell colony formation assay, cells were plated into six-well plates (500 cells/well). After 2 weeks, the cells were washed, fixed and then stained with 0.1% crystal violet, and cell colonies that grew to over 50 cells were counted.

### CCK-8 assay

A cell counting kit-8 (Biosharp, BS350B) was used to detect cell viability after being treated with cisplatin (0.6 μM). Cells were cultured in 96-well plates (3000 cells/well). The working solution consisting of CCK-8 reagent and DMEM medium was added to the well (100 mL/well). After being incubated for 3 h, the absorbance of the solution at 450 nm was measured.

### EdU assay

The EdU assay was carried out using an EdU-594 kit (Beyotime, C0078S, China) according to the instruction manual. Briefly, cells were plated into 96-well plates (4 × 10^4^ cells/well) in triplicate. After the cells adhered, they were maintained in the presence of 10 mM EdU in DMEM for 2 h and then fixed with 4% paraformaldehyde for 15 min. The cells were permeabilized and then incubated with Azide-594 mixture for 30 min to facilitate a click reaction and then with Hoechst 33342 to stain the nuclei for 10 min. The cells were viewed and images were captured for analysis.

### Wound-healing assay

Cells were cultured in six-well plates until they formed a free-of-void monolayer. The monolayers were then scratched using pipette tips to generate wounds. Detached cells were washed with PBS. Then, the cells were further cultured in serum-free DMEM. Wounds were photographed at 0 h and 72 h using a light microscope, and the migration rate was calculated using ImageJ software.

### Cell migration assay

Cells were collected and washed with serum-free DMEM three times and then seeded into the upper chamber (6 × 10^4^ cells/chamber) in three replicates. Cells growing in the upper chamber were cultured in serum-free DMEM. DMEM supplemented with 10% FBS was added to the lower chamber to induce cell migration. After 24 h, the cells were fixed and dyed with 0.1% crystal violet. The migrated cells were observed and images were captured to count the cells.

### Cell cycle analysis

Cells that underwent siRNA transfection were subjected to 6 Gy IR exposure and allowed to recover from the irradiation for 4 h. Subsequently, the cells were suspended and fixed with 70% ethanol overnight at 4 °C, followed by propidium iodide (PI) staining for 30 min at room temperature. Then, the cells were detected by flow cytometry, and the cell cycle was analyzed using ModFit software.

### Immunofluorescence staining

Cells were grown on sterilized coverslips and subjected to 6 Gy IR exposure. After 4 h, the cells were fixed with 4% paraformaldehyde for 15 min and permeabilized with 0.2% Triton X-100 for 15 min. After blocking for 1 h, the cells were incubated with γ-H2AX (Abcam, ab26350, 1:1000) and Rad51 (Abcam, ab133534, 1:1000) antibodies overnight and the corresponding secondary antibody for 1 h. DNA was stained with DAPI for 10 min. Cell images were acquired with a confocal fluorescence microscope, and cells with over 10 foci were considered to be positive.

### Neutral comet assay

To assess DNA damage in individual cells, a comet assay kit (Trevigen, 4250-050-K, USA) was used. Huh7 and HepG2 cells were subjected to 6 Gy IR exposure and were collected 4 h post-irradiation. Cells and molten low-melting agarose (LMA) were combined, and the mixture was evenly coated onto comet slides. Then, the cells were lysed for 1 h and immersed in neutral electrophoresis buffer and incubated for 0.5 h before electrophoresis for 1 h at 4 °C. The slides were then immersed in DNA precipitation solution and incubated for 0.5 h, after which samples were stained with SYBR Gold at room temperature. The Olive tail moment was analyzed using CometScore2.0 and GraphPad Prism software, and at least 100 cells were included.

### Cell clonogenic survival assay

This assay was performed as previously described [35]. Cells were plated in six-well plates in triplicate at densities of 200, 400, 1000, 4000, and 8000 cells/well and irradiated at 0, 2, 4, 6, and 8 Gy. Three days after irradiation, the cell medium was removed, fresh medium was added, and the cells were cultured for two weeks. The cells were then fixed and stained, and the colony number (clusters of >50 cells) of each treatment group was calculated.

### Animal experiment

Nude mice 4–6 months of age were randomly divided into two groups and then injected subcutaneously with 5 × 10^6^ cells of Sh-Control or Sh-KDM4C HepG2 stable cells. Mice were fed in aseptic conditions and the long diameter (a) and short diameter (b) of the tumors were measured every 3 days. The formula V = a × b^2^/2 was used to calculate the tumor volume. The mice were ultimately euthanized and the tumor weight was measured. The animal experiment was approved by the Medical Ethics Committee of Tongji Medical College, Huazhong University of Science and Technology.

### Statistical analysis

Every experiment we performed was repeated at least three independent times. Statistical analysis of group comparisons was performed using Student’s *t*-test (two-tailed). *P* < 0.05 was considered statistically significant. *P < 0.05, **P < 0.01, ***P < 0.001.

## Supplementary information

Supplementary Table 1

Supplementary Table 2

Full and uncropped western blots

## Data Availability

The datasets used and/or analyzed during the current study are available from the corresponding author on reasonable request.
