# Supplementary material for: KDM4C silencing inhibits cell migration and enhances radiosensitivity by inducing CXCL2 transcription in hepatocellular carcinoma
Source: Cell Death Discov. 2023 Apr 28;9:137. doi: 10.1038/s41420-023-01418-w (PMC10147924; doi:10.1038/s41420-023-01418-w)
Supplement: Supplementary file 1 — Supplementary Table 1 [file 41420_2023_1418_MOESM1_ESM.docx]

**Supplementary Table 1.** Primer Sequences used for qRT-PCR

| Genes | Sequences (5'-3') |
| --- | --- |
| CXCL2 | F: TGCTCCTGCTCCTGGTGG  R: TGCCCATTCTTGAGTGTGGC |
| KLF2 | F: CTGCCGTCCTTCTCCACTTT  R: AGGATGAAGTCCAGCACGCT |
| FGF18 | F: GCGAGGATGGGGACAAGTAT  R: GCGGTTCATGCACAGGTAGA |
| HKDC1 | F: CTGGCACCAATGCGTGTTAC  R: CAGTTGCTTTCCTGGGTTGAG |
| WNT6 | F: TATGGACCCTACCAGCATCTG  R: GGAACTGGAACTGGCACTCT |
| KDM4C | F: CATCGGAACACCCGGTATTAC  R: TTCAGACAGTCTCGGCTCAC |
| GAPDH | F: AGAAGGCTGGGGCTCATTTG  R: AGGGGCCATCCACAGTCTTC |

F, forward primer; R, reverse primer.
