# Supplementary material for: KDM4C silencing inhibits cell migration and enhances radiosensitivity by inducing CXCL2 transcription in hepatocellular carcinoma
Source: Cell Death Discov. 2023 Apr 28;9:137. doi: 10.1038/s41420-023-01418-w (PMC10147924; doi:10.1038/s41420-023-01418-w)
Supplement: Supplementary file 2 — Supplementary Table 2 [file 41420_2023_1418_MOESM2_ESM.docx]

**Supplementary Table 2.** Primer Sequences used for ChIP-PCR

| Gene | Primers | Sequences (5'-3') |
| --- | --- | --- |
| CXCL2 | Primer 1 | F: AACGGCGAACCCCTTTTATG  R: GGCAGAAAGAGAACATCCCAC |
|  | Primer 2 | F: GGCGAACCCCTTTTATGCAT  R: CGGGGCAGAAAGAGAACATC |
|  | Primer 3 | F: GGGTCTGACTGTCTTGCGTA  R: GGCGGTTATCTCGGTATCTCT |

F, forward primer; R, reverse primer.
