# Supplementary figures and images for: KDM4C silencing inhibits cell migration and enhances radiosensitivity by inducing CXCL2 transcription in hepatocellular carcinoma
Source: Cell Death Discov. 2023 Apr 28;9:137. doi: 10.1038/s41420-023-01418-w (PMC10147924; doi:10.1038/s41420-023-01418-w)

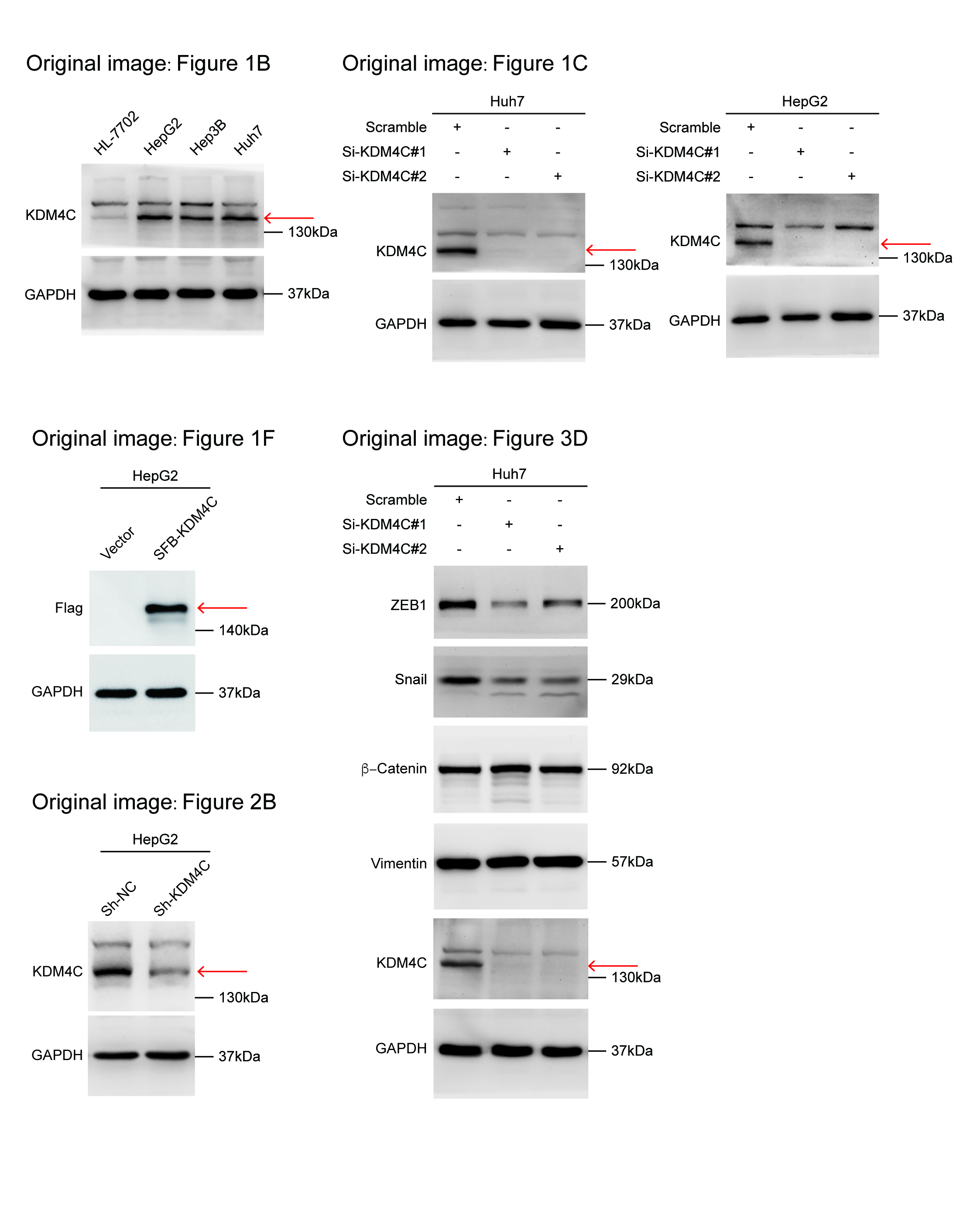

Supplement: Supplementary file 3 — Full and uncropped western blots [file 41420_2023_1418_MOESM3_ESM.tif]
